# Thioredoxin from the Indianmeal Moth *Plodia interpunctella*: Cloning and Test of the Allergenic Potential in Mice

**DOI:** 10.1371/journal.pone.0042026

**Published:** 2012-07-26

**Authors:** Elisabeth Hoflehner, Marina Binder, Wolfgang Hemmer, Vera Mahler, Raphael C. Panzani, Reinhart Jarisch, Ursula Wiedermann, Michael Duchêne

**Affiliations:** 1 Institute of Specific Prophylaxis and Tropical Medicine, Center for Pathophysiology, Infectiology and Immunology, Medical University of Vienna, Vienna, Austria; 2 Floridsdorfer Allergiezentrum, Vienna, Austria; 3 Department of Dermatology, University of Erlangen-Nuremberg, Erlangen, Germany; 4 Centre de recherche en allergologie, Marseille, France; University Paris Sud, France

## Abstract

**Background/Objective:**

The Indianmeal moth *Plodia interpunctella* is a highly prevalent food pest in human dwellings, and has been shown to contain a number of allergens. So far, only one of these, the arginine kinase (Plo i 1) has been identified.

**Objective:**

The aim of this study was to identify further allergens and characterise these in comparison to Plo i 1.

**Method:**

A cDNA library from whole adult *P. interpunctella* was screened with the serum of a patient with indoor allergy and IgE to moths, and thioredoxin was identified as an IgE-binding protein. Recombinant thioredoxin was generated in *E. coli*, and tested together with Plo i 1 and whole moth extracts in IgE immunoblots against a large panel of indoor allergic patients' sera. BALB/c mice were immunised with recombinant thioredoxin and Plo i 1, and antibody production, mediator release from RBL cells, T-cell proliferation and cytokine production were measured.

**Result:**

For the first time a thioredoxin from an animal species was identified as allergen. About 8% of the sera from patients with IgE against moth extracts reacted with recombinant *P. interpunctella* thioredoxin, compared to 25% reacting with recombinant Plo i 1. In immunised BALB/c mice, the recombinant allergens both induced classical Th2-biased immune responses such as induction IgE and IgG1 antibodies, upregulation of IL-5 and IL-4 and basophil degranulation.

**Conclusion:**

Thioredoxin from moths like Plo i 1 acts like a classical Type I allergen as do the thioredoxins from wheat or corn. This clearly supports the pan-allergen nature of thioredoxin. The designation Plo i 2 is suggested for the new *P. interpunctella* allergen.

## Introduction

Arthropods represent more than three quarters of all animal species, and get into contact with humans in multiple ways. Arthropod antigens can cause Type I allergies with various manifestations, such as the acute and life-threatening insect venom allergies [1], perennial rhinitis, dermatitis and bronchial asthma caused by house dust mites [2] or cockroaches [3], [4], as well as oral symptoms caused by seafood [5].

Although moths like cockroaches are typical and widespread household pests, there is much less research on Type I allergy against moth proteins. Historically, that moths cause inhalant allergies had been reported as early as 1928 by Vaughan [6]. In the following decades, there have been occasional case reports on bronchial asthma caused by moths, such as by the clothes moth *Tineola bisselliella* in an infested home [7] or the wax moth *Galleria mellonella* in a company producing fish bait [8]. More recently, IgE immunoblots of clothes moth [9] or silkworm moth [10] demonstrated specific IgE against moth antigens.

The Indianmeal moth *Plodia interpunctella* is a common household and stored product pest. Its larvae feed on dry foodstuffs such as grains, nuts, dried fruit, or chocolate. This pest had been suspected early to be a possible source of allergens in mills [11]. The first *P. interpunctella* allergen to be identified on the molecular level (Plo i 1) was an arginine kinase [12]. Arginine kinase (EC 2.7.3.3) phosphorylates arginine, whereas the related creatine kinase (EC 2.7.3.2) acts on creatine. Both enzymes thus help to store metabolic energy, where creatine kinase is typically found in vertebrates and arginine kinase is found in lower species such as arthropods and some protozoa [13]. The arthropod arginine kinases exhibit IgE cross-reactivity [12], and several further arginine kinases have been identified as allergens, *e.g.* from shrimp [14], [15] and mite [16].

IgE immunoblots of *P. interpunctella* whole larval extracts were positive in 51% when probed with sera from indoor allergic patients [12]. In this study we extend this work using extracts from adult moths. IgE screening of a cDNA library of the imago stage of *P. interpunctella* revealed a further allergen, a thioredoxin.

Thioredoxins are ubiquitous redox-active proteins found in eukaryotes and prokaryotes. They activate proteins by reduction of cysteine disulphide bonds [17], [18]. Thioredoxins have been identified as allergens in wheat and maize [19] as well as fungi such as *Malassezia* spp. [20]. In human allergic bronchopulmonary aspergillosis, IgE cross-reactivity of fungal and human thioredoxin was found to result in IgE autoreactivity [21]. Recently, wheat flour thioredoxin was identified as an allergen in patients with baker's asthma [22].

In this study, the allergenic potential of thioredoxin as a new arthropod allergen was examined in comparison to Plo i 1 by IgE immunoblotting and in a mouse immunisation model.

## Materials and Methods

### Ethics statement and patient characteristics

The study was approved by the ethics committee of the Medical University of Vienna and the Vienna General Hospital (application EK Nr. 322/2008). The anonymised sera to be tested in immunoblots (n = 156, of which n = 154 were allergic patients'sera and n = 2 nonallergic control sera) are displayed in detail in Table 1.

**Table 1:** Demographics and symptoms of the groups of patients' sera examined in this study.

Symptomatic Type I allergy against	Abbre-viation in Fig. 1	Number of patients	Age range	Mean age	Female∶ male ratio	RC [%]	Asthma [%]	AD [%]
House dust mites but not animal dander	M	69	1–64	21.3	31 ∶ 38	77	38	6
House dust mites and animal dander	M+D	43	9–61	30.0	16 ∶ 27	74	49	12
Animal dander but not house dust mites	D−M	20	3–56	27.7	10 ∶ 10	60	65	0
Seafood	SF	22	17–76	38.5	7 ∶ 15	50	36	0
Nonallergic controls	NC	2	37–49	44.0	1 ∶ 1	0	0	0
Total allergy patients		154	1–76	27.0	64 ∶ 90	70	44	6
Total individuals		156	1–76	27.2	65 ∶ 91			

The sera were from Floridsdorfer Allergiezentrum, Vienna, Austria. The last three colums give the percentage of patients reporting rhinitis and/or conjuctivitis (RC), asthma or asthma-like symptoms (“asthma”), or atopic dermatitis (AD).

The diagnosis of Type I allergy was based on case history and symptoms (rhinitis/conjunctivitis, allergic asthma), skin prick testing as well as CAP-RAST (Pharmacia, Uppsala, Sweden) testing using a panel of extracts from indoor (house dust mite, cat dander) and outdoor (birch pollen, grass pollen) allergen sources. The cDNA library from adult *P. interpunctella* was screened with the serum from an indoor-allergic patient with IgE reactivity to Indianmeal moths.

### Construction of a *P. interpunctella* cDNA library from imagines

*P. interpunctella* larvae were left to hide for metamorphosis in a roll of cardboard with vertical holes. When the moths emerged, they were immediately frozen in liquid nitrogen. Four hundred moths (around 3.2 g) were homogenised in 30 ml of TRIzol reagent (Life Technologies, Frederick, MD), and about 10 mg of total RNA were extracted. Poly(A)^+^ RNA was prepared using the Poly(A)Ttract system (Promega, Madison, WI, USA) with a yield of 4.9 µg from 4 mg of total RNA. The cDNA library prepared with the Uni-ZAP system (Stratagene, La Jolla, CA, USA) [23] contained 1.25×10^6^ clones.

### IgE screening of the λZAP cDNA library

λZAP phages (2.8×10^5^) from the primary library were plated on *Escherichia coli* XL1-Blue (Stratagene). Synthesis of recombinant proteins was induced by adding nitrocellulose filters (Schleicher & Schuell, Dassel, Germany) soaked in 10 mM isopropylthio β-D-galactoside (IPTG). The filters were blocked, and probed with a 1/10 dilution of a serum which contained IgE against several moth proteins. IgE bound to recombinant moth proteins was detected by ^125^I-labeled anti-IgE antibodies (Pharmacia) followed by autoradiography. Further rounds of screening were then performed to obtain single phage clones.

The cDNA-containing plasmids were obtained from the isolated phages by *in vivo* excision [23]. Twenty two cDNAs were sequenced using Thermosequenase (Amersham Pharmacia Biotech, Piscataway, NJ, USA) and IRD800-labeled primers on a LI-COR sequencer (LI-COR, Lincoln, NE, USA). The deduced protein sequences were compared with the SwissProt database using the FastA program [24]. Nucleotide sequences were aligned with the ClustalW program [25].

### Expression of *P. interpunctella* thioredoxin in *E. coli*

The oligodeoxynucleotide primers ExTrxF 5′-GGG ACT TC**C ATA TG**T CGA TCC ACA TCA AAG ACG TTG AGG AC-3′ and ExTrxR 5′-TCC G**CT CGA G**TT AAT GAT GGT GAT GAT GAT GCT TGA GTT TCA AAA TGG TGG ACC G-3′ were used for PCR to amplify the *P. interpunctella* thioredoxin coding sequence from the cDNA template (clone 1). The primers contain *Nde*I and *Xho*I restriction sites (bold) for cloning into the expression plasmid pET-17b (Novagen, Merck, Darmstadt, Germany) plus a hexahistidine-encoding sequence (underlined) for recombinant protein purification.

The recombinant pET-17b plasmid was transformed into *E. coli* BL21-CodonPlus (DE3) (Stratagene). The cultures were grown to an OD_600_ of 0.75, recombinant protein production was induced by addition of 1 mM IPTG and shaking overnight at 18°C. Harvesting of cells, lysis and purification of recombinant thioredoxin via metal chelate affinity chromatography under native conditions were performed according to the supplier's protocol (Qiagen, Hilden, Germany). Recombinant *P. interpunctella* arginine kinase (Plo i 1) was produced as described [12].

### IgE-immunoblots of moth extracts and the recombinant allergens

Whole extracts from imagines of the Mediterranean flour moth (*Ephestia kuehniella*), a species closely related to *P. interpunctella* but considerably larger, were obtained by grinding of 150 moths with liquid nitrogen in a mortar. Fifteen ml of 1× reducing gel loading buffer were added, samples were denatured for 10 min at 95°C, and debris was removed by centrifugation. The whole extracts or, respectively, the purified recombinant allergens (arginine kinase (Plo i 1) and moth thioredoxin) were separated on preparative 12.5% SDS - polyacrylamide gels with an approximate protein concentration of 20 µg/cm (extracts) or 10 µg/cm (of each purified recombinant protein) as estimated by Coomassie blue-stained test gels. Proteins were blotted onto nitrocellulose membranes (Schleicher & Schuell), and 5 mm strips were cut after the transfer. The strips were blocked 2×5 min and 1×30 min at room temperature with buffer G (42 mM Na_2_HPO_4_, 6.4 mM NaH_2_PO_4_, 0.5% (v/v) Tween 20, 0.5% (w/v) bovine serum albumin, 0.05% (w/v) NaN_3_, pH 7.5), and incubated with a 1/10 dilution of patients' sera in buffer G overnight at 4°C. After washing 2×5 min and 1×30 min in buffer G, bound IgE was detected by overnight incubation at room temperature with ^125^I-labeled anti-IgE antibodies (IBL, Hamburg, Germany), washing as above, and autoradiography at −80°C.

### Ethics statement on animal treatment and sampling

The animal study was performed according to institutional guidelines for animal use and care. The study was approved under the number GZ BMBWK-66.009/0246-BrGT/2005 by the Austrian Federal Ministry of Science and Research.

Female BALB/c mice (n = 5 per group, aged 7 weeks) were purchased from the Research Institute for Laboratory Animal Breeding (Himberg, Austria). Sensitisation was performed by four subcutaneous (s.c.) injections (day 0, 14, 28, 42) of 1 µg, 5 µg or 25 µg of Plo i 1 (arginine kinase) or Plo i 2 (thioredoxin) adsorbed to aluminium hydroxide (Al(OH)_3_; Serva, Heidelberg, Germany). Control mice were sham-treated with PBS. Blood samples were taken before treatment (day 0), and 7 days after the last immunisation (day 49) by tail bleeding. At sacrifice cell suspensions from spleens were prepared as described [26].

### Allergen-specific antibody levels in mouse sera

Microtiter plates (Nunc, Roskilde, Denmark) were coated with 5 µg/ml of the recombinant allergens Plo i 1 or Plo i 2, respectively, and incubated with sera. Serum samples were diluted 1/500 for IgG2a, 1/1000 for IgG1 and 1/10 for IgE. Rat anti-mouse IgG2a, IgG1 and IgE antibodies (1/500, Pharmingen, San Diego, CA) were used, followed by peroxidase-conjugated mouse anti-rat IgG antibodies (1/2000, Jackson Immuno Lab, West Grove, PA) [26]. Data are shown as optical density (OD) after subtraction of baseline levels from pre-immune sera.

### Rat basophil leukaemia cell mediator release assay (RBL assay)

Sera were incubated with RBL-2H3 cells (cell line from rat basophilic leukemia, available from ATCC, No. CRL-2256) at dilutions of 1/10. IgE-dependent degranulation of RBL cells was induced by adding 0.03 µg of each allergen (Plo i 1 or Plo i 2) diluted in 100 µl Tyrode's buffer. Supernatants were analysed for ß-hexosaminidase activity as described previously [27]. Data are reported as percen-tages of total ß-hexosaminidase released after addition of 1% Triton X-100 and are shown after subtraction of baseline release levels obtained with pre-immune sera.

### Lymphocyte proliferation and cytokine production

Proliferation of splenocytes (2×10^5^ cells/well) after stimulation with the recombinant allergens Plo i 1 or, respectively, Plo i 2 (2 µg/well) was measured as described previously [27]. The ratio of the mean proliferation after antigen stimulation (counts per minute [cpm]) and medium control values [cpm], *i.e.* the stimulation index [SI], was calculated.

Levels of IL-4, IL-5 and IFN-γ were measured in spleen cell cultures (5×10^6^ cells/well) incubated for 48 hours with Plo i 1 or Plo i 2 (10 µg/well) [26]. Cytokine levels were measured with mouse ELISA kits (Endogen, Woburn, MA) and were shown in pg/ml after subtraction of baseline levels of unstimulated cultures.

### Statistics

Data are expressed as means ± SEMs. For statistical analysis p values<0.05 were defined significant. Pair-wise comparison of allergen- sensitised versus sham-treated control groups was performed by using the non-parametric Kruskal–Wallis test, followed by ad hoc post analysis.

## Results

### Prevalence of IgE binding to moth extracts in sera from indoor-allergic patients

Previously we had shown that 51% of unselected patients with indoor allergies had serum IgE antibodies against *P. interpunctella* larval extracts. In the present study we tested 154 anonymised type I allergic patients' sera and two non-allergic control sera from Vienna (see Table 1). These comprised the following groups of patients: patients with indoor allergies to house dust mite (n = 69), to house dust mite and animal dander (n = 43), to animal dander but not house dust mite (n = 20), in addition seafood allergic patients (n = 22), and two non-allergic control sera.

**Figure 1:**
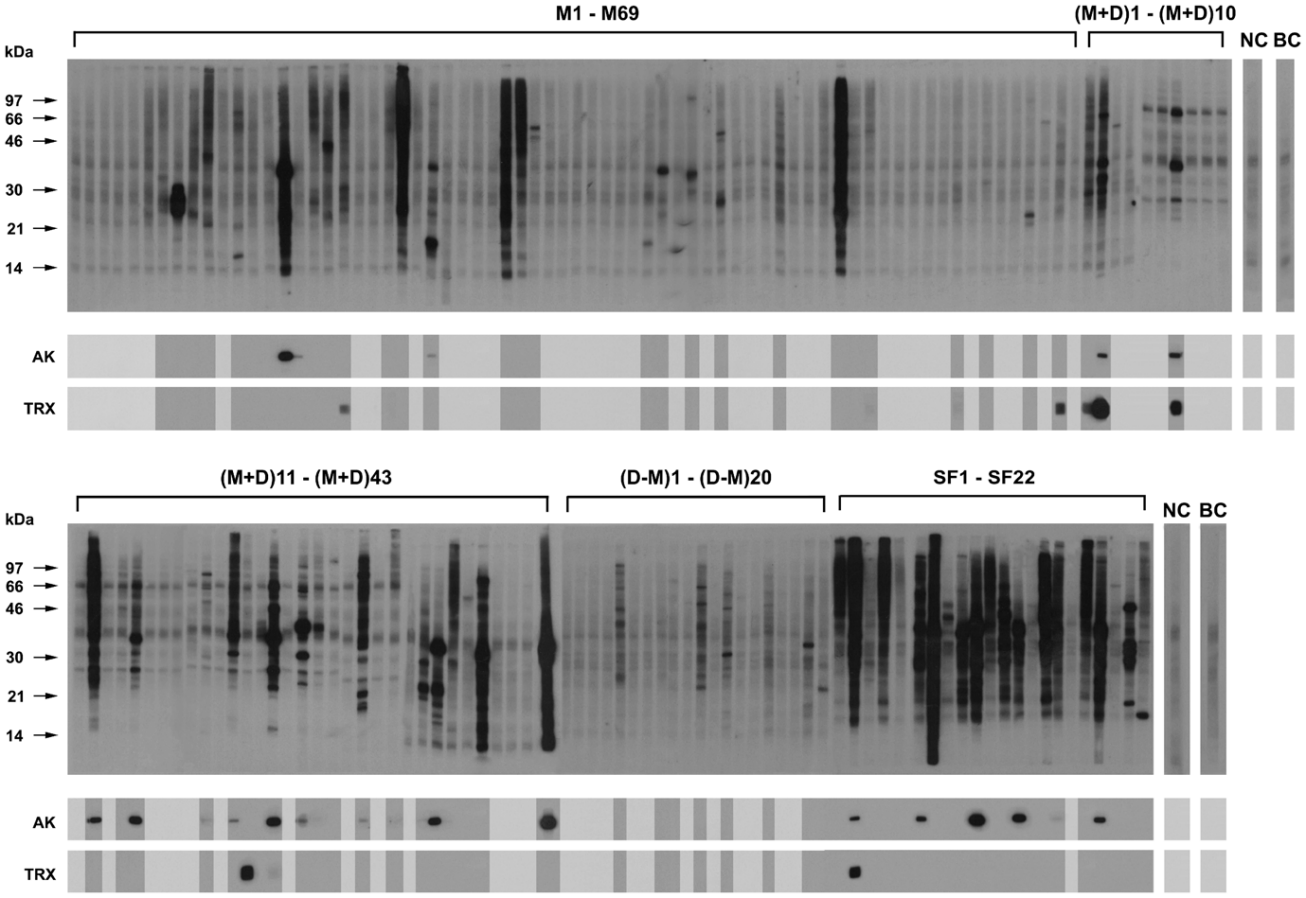
IgE immunoblots with patients' sera. Top panels: extracts from whole mediterranean flour moths (*E. kuehniella*) were separated by polyacrylamide electrophoresis, blotted onto nitrocellulose, and probed with the sera from various groups of patients with indoor allergy or seafood allergy as well as from control individuals. The indoor allergic patient groups were: M, housedust mite allergic patients (n = 69), M+D, patients allergic to mites and animal dander (n = 43), D−M, patients with allergy to animal dander but not to mites (n = 20). Patients SF, seafood allergic patients (n = 22), NC, nonallergic control individuals (n = 2), BC, buffer control without serum, only secondary anti-IgE antibody. Molecular weights [kDa] are indicated at the left side. Bottom panels, the recombinant *P. interpunctella* 40 kDa arginine kinase (AK) and 12 kDa thioredoxin (TRX) were probed with those patients' sera containing IgE to *E. kuehniella*, and only the parts of the strips containing the two recombinant allergens are shown.

Extracts from whole *E. kuehniella* moths, flour moths closely related to *P. interpunctella*, but yielding significantly more extract per animal, were used to prepare immunoblot strips. These strips were used to probe all the sera for IgE antibodies against moth antigens. The results are shown in Fig. 1 (top parts). A low IgE reactivity, which we interpret as weak binding of the moth antigens to the secondary antibody, was observed in the sera from nonallergic individuals (NC) and the buffer controls (BC) and is always taken into account. In the indoor allergic patient groups, 48% the sera reacted significantly with moth allergens contained in the whole extract, similar to the 51% found previously for *P. interpunctella* larval antigens [12]. This varied from 43% in the group of indoor allergic patients with mite allergy only (M) to 53% in the patients with allergies to mites and animal dander (M+D). In the group with indoor allergy to animal dander but not to mites (D−M), 50% reacted with moth allergens. Although the frequency of IgE recognition did not vary so much between the groups, a striking difference was observed in the quantity of moth specific IgE. In a number of the mite allergic and moth IgE-positive patients, and even more in the mite and dander allergic and moth IgE-positive patients, very high moth specific IgE levels were observed. None of the 20 patients tested negative for mites (D−M) showed such a high level of reactivity. The binding of IgE to moth antigens was highest, however, in the group of seafood allergic patients (SF), in relative numbers, 95% of the sera were positive, and 55% of these sera contained very high levels of levels of IgE binding to moth antigens.

Taken together, immunoreactive bands of various molecular weights were observed on the blots and in order to identify more moth allergens, a moth cDNA library was constructed and screened.

### Cloning of *P. interpunctella* thioredoxin as an IgE-binding protein from adult moths

As described above, a λZAP cDNA library from *P. interpunctella* imagines was probed with the serum from a patient with indoor allergies and IgE binding to several moth antigens. The primary screen gave 40 strong and 10 weak positive signals. After reprobing, 22 single cDNA clones were obtained and sequenced. Twenty of these encoded a *P. interpunctella* protein with significant sequence similarity to thioredoxins. Three of the 20 clones contained single base insertions and deletions resulting in truncation of the open reading frame. The other 17 clones comprised the identical open reading frame encoding a protein of 11.7 kDa. The 3′-untranslated region of 689 bp between the stop codon and the poly-A tail was rather long. Minor differences were obtained in this region, but these did not allow to group the cDNAs into possible isoforms. The complete sequence of clone 1 was submitted to the EMBL/GenBank/DDBJ databases (accession number FR681573).

**Figure 2:**
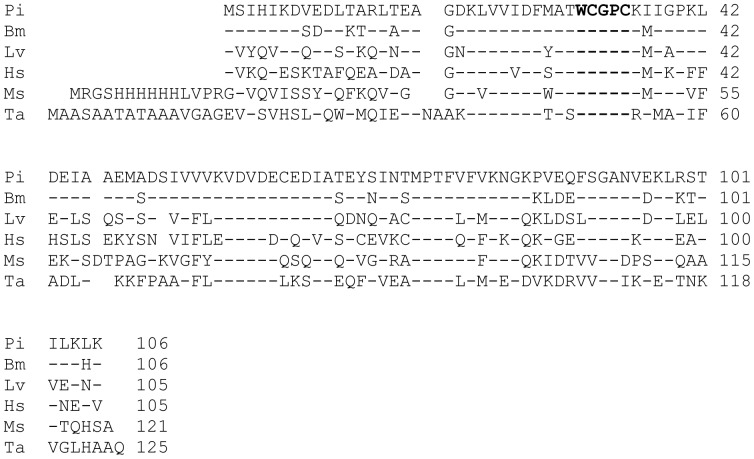
Comparison of thioredoxin sequences. The deduced amino acid sequence of *P. interpunctella* thioredoxin Plo i 2 (Pi) was aligned with *Bombyx mori* thioredoxin (ABM92269) (Bm) with 83% identity. The other arthropod thioredoxin from the shrimp *Litopenaeus vannamei* (ACA60746) (Lv) was 58% identical, and human thioredoxin (NP_003320) (Hs) shared 47% amino acid identity with Pi. The allergenic fungal thioredoxin (2J23_A) Mala s 13 from *Malassezia sympodialis* (Ms) and wheat (*Triticum aestivum*) thioredoxin Tri a 25 (Ta) (CAB96931) were 46% and 41% identical, respectively with Plo i 2. The residues equal to Plo i 2 are marked with dashes. The important thioredoxin consensus sequence WCGPC with the two active site cysteines was found in all the sequences (bold type).

The BlastP search clearly identified the protein as thioredoxin. Fig. 2 shows *P. interpunctella* thioredoxin (Pi) aligned with selected other thioredoxins. The most closely related sequence with 83% amino acid identity was from the domestic silkworm *Bombyx mori* (Bm). The thioredoxin from the more distantly related arthropod *Litopenaeus vannamei* (Lv) and human thioredoxin (Hs) were 58% and 46% identical, respectively, to *P. interpunctella* thioredoxin. The allergenic fungal thioredoxin (2J23_A) Mala s 13 from *Malassezia sympodialis* (Ms) and wheat (*Triticum aestivum*) thioredoxin Tri a 25 (Ta) (CAB96931) were 46% and 41% identical. All the sequences contained the active site consensus WCGPC with the two redox-active cysteines.

We obtained only two other IgE-positive cDNAs from the same screen not coding for thioredoxin. The delicate extraction of RNA from adult moth material, a possible advantage of λZAP phage expressing thioredoxin leading to a preponderance in the composition of the plated library, or a peculiarity of the patient's serum used for screening may contribute to this finding. We did not investigate this matter in more detail and focused on the characterisation of thioredoxin as a novel allergen from moths.

### Prevalence of IgE binding to recombinant *P. interpunctella* thioredoxin and arginine kinase (Plo i 1) in sera from indoor-allergic patients

Recombinant thioredoxin (11.7 kDa) and arginine kinase, Plo i 1, (39.9 kDa) [12] from *P. interpunctella* were separated together under denaturing and reducing conditions in SDS-PAGE, blotted onto nitrocellulose, and strips were probed with the sera that had shown positive reactivity with the whole moth extracts. The signals from the recombinant allergens arginine kinase Plo i 1 and thioredoxin are displayed in Fig. 1 as two blocks AK (40 kDa) and TRX (12 kDa) mounted below the whole moth extract strips.

Counting only the clearly positive signals, seven patients' sera had IgE towards *P. interpunctella* thioredoxin, six in the various indoor allergic groups and one in the seafood allergic group, but none in mite negative group. This corresponds to 8% of all moth extract positive patients, 5% of all allergic patients of this study. Again the strongest signals were observed in patients with concomitant mite allergy or concomitant mite and dander allergy, and in the seafood allergic patients. Due to this small but significant number of sera with IgE to moth thioredoxin, we proposed to name this allergen Plo i 2, and it is now available in the IUIS allergen nomenclature database as Plo i 2.0101.

**Figure 3:**
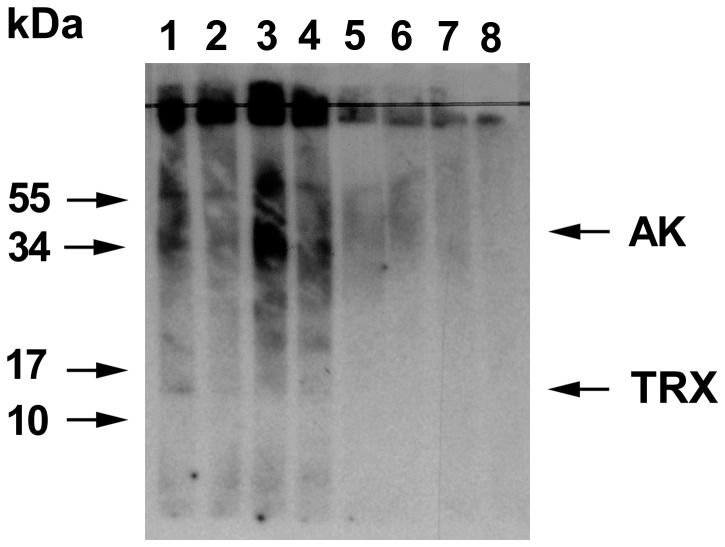
Immunoblot inhibition experiment. Extracts from *E. kuehniella* were separated by polyacrylamide electrophoresis, blotted onto nitrocellulose, and strips were probed with serum samples preincubated with or without 10 µg of recombinant allergen overnight. Lane 1, thioredoxin (Plo i 2) positive serum; lane 2, same serum preincubated with recombinant *P. interpunctella* thioredoxin; lane 3, arginine kinase (Plo i 1) positive serum; lane 4, same serum preincubated with recombinant Plo i 1; lane 5, nonallergic control serum, lane 6, same serum preincubated with Plo i 2; lane 7, same serum preincubated with Plo i 1; lane 8, buffer control. Molecular weights [kDa] are indicated at the left side, the positions of thioredoxin (TRX) and arginine kinase (AK) at the right side.

In contrast to the Plo i 1 homolog, we noted the absence of 12 kDa bands of the natural thioredoxin in the blots from the whole moth extracts. This could have several causes, for example, the concentration of thioredoxin could simply be low, or as the immunoblots for whole moth extracts were not optimised for small proteins, some thioredoxin might have been lost during blotting. Alternatively, thioredoxin still bound covalently to its interaction partners [17], [18] could migrate at higher molecular weights. Finally, the patients' IgE could possibly recognise *P. interpunctella* thioredoxin better than the *E. kuehniella* homolog. To investigate this cross-reactivity, we performed an inhibition experiment as done previously [12] (Fig. 3). Two 1 ml samples of a 1∶10 dilution of a thioredoxin positive serum in buffer G (see Materials and Methods) were preincubated with or without 10 µg of recombinant *P. interpunctella* thioredoxin overnight at 4°C, and then used to probe strips of blotted *E. kuehniella* antigens as described above (Fig. 3, lanes 1, 2). Similarly an inhibition was carried out with a Plo i 1 positive serum and recombinant Plo i 1 (Fig. 3, lanes 3, 4) or a nonallergic control serum (Fig. 3, lanes 5–7), here lane 5 shows the uninhibited serum, lane 6 was preincubated with recombinant thioredoxin and lane 7 with recombinant arginine kinase. Lane 8 represents the buffer control. One important result of the experiment was that the 12 kDa thioredoxin band only appeared after the blot was exposed for 14 days instead of two days. This shows that the extracts contained only a low concentration of thioredoxin and explain the absence of signals in the previous blots. With the help of specific antibodies against recombinant Plo i 2, it should be possible to better quantify the allergen in the extracts and to check if it forms covalent adducts of larger molecular weight with its interaction partners.

The main result of the inhibition experiment was that recombinant Plo i 2 was able to block the binding of the patient's IgE to the *E. kuehniella* thioredoxin (Fig. 3, lane 2) as well as recombinant Plo i 1 blocked the binding to *E. kuehiella* arginine kinase (Fig. 3, lane 4). So the allergens from the closely related moths are cross-reactive.

The immunoblot experiment with the recombinant arginine kinase (Fig. 1, lower panels) showed that 21 of all the patients with IgE to moth extracts (25%) had specific IgE to Plo i 1, and again the groups with mite, mite and dander, and seafood allergies had the highest prevalence and displayed the strongest signals, no Plo i 1 positive reactions were detected in any mite negative patient. The prevalence of IgE to Plo i 1 is the same as the 25% frequency of recognition of Plo i 1 in moth larval extract positive patients observed previously [12].

### Antibody production in sensitised mice

**Figure 4:**
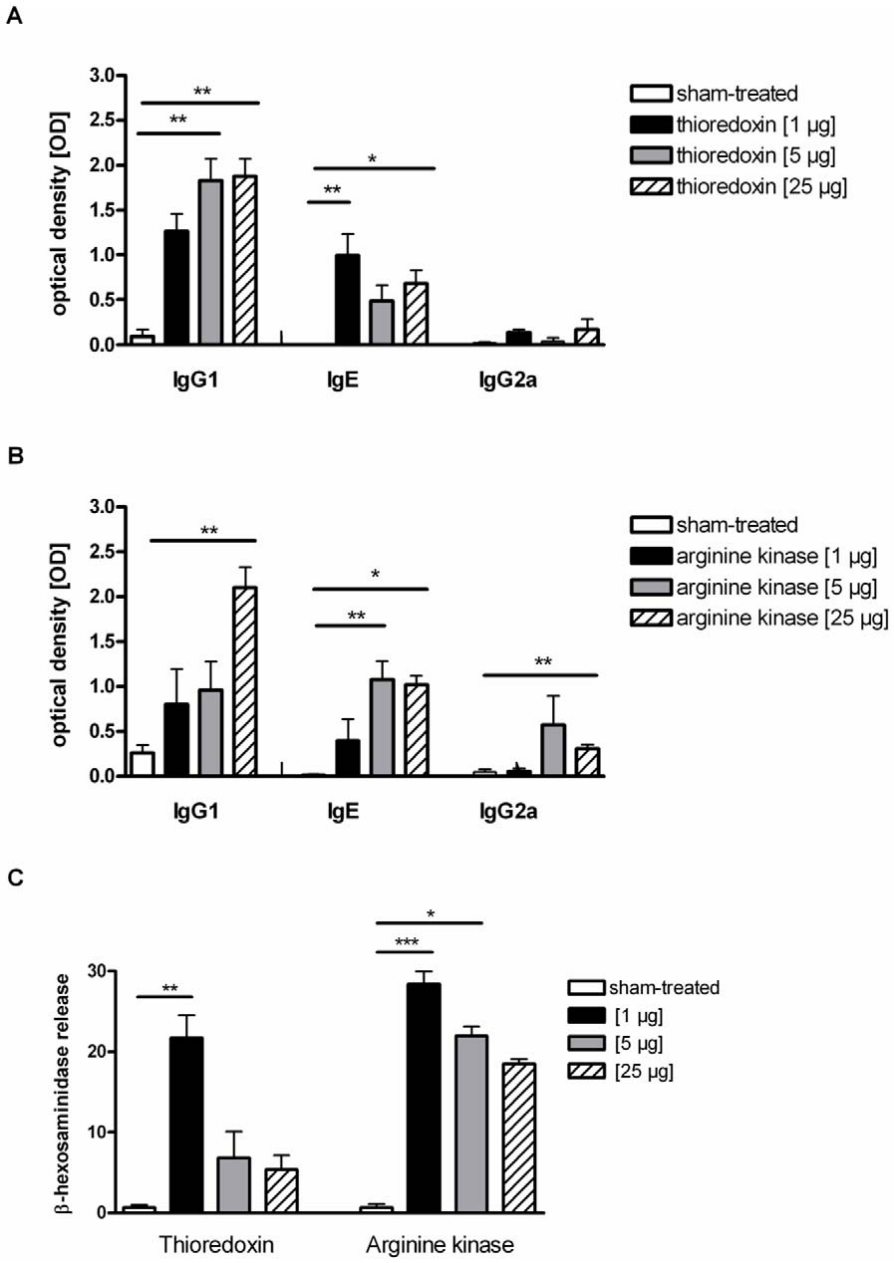
Antibody levels in mouse sera. (A), thioredoxin, (B), arginine kinase-specific antibody responses in sera after immunisation with three different antigen concentrations (1 µg, 5 µg, 25 µg) measured by ELISA; (C), IgE-mediated basophil degranulation in sera, β- hexosaminidase release from thioredoxin- and arginine kinase-sensitised mice, treated with 1 µg (black bars), 5 µg (grey bars) or 25 µg (striped bars), compared to sham-treated controls (white bars). *p<0.05, **p<0.01, ***p<0.005.

To investigate the immunogenicity of thioredoxin (Plo i 2) and arginine kinase (Plo i 1) we sensitised mice four times subcutaneously with three different concentrations of the recombinant allergens (1 µg, 5 µg, 25 µg). Treatment with thioredoxin induced strong Th2 responses with increased IgG1 levels for all three concentrations, whereas treatment with 5 µg and 25 µg showed significantly increased antibody levels in comparison to sham-treated mice. IgE antibody production in thioredoxin-treated mice was significantly upregulated after sensitisation with the lowest concentration of 1 µg and with the highest concentration of 25 µg allergen, respectively, compared to controls. The typical Th1-associated IgG2a was only enhanced in mice, treated with 1 µg and 25 µg thioredoxin (Fig. 4A).

Arginine kinase-specific IgG1 was induced with all three concentrations, but only treatment with 25 µg showed a significantly increased antibody production, compared to controls. Whereas arginine kinase-specific IgE was significantly induced with a concentration of 5 µg as well as 25 µg, in comparison to sham-treated mice, IgG2a production was significantly increased in 25 µg- arginine kinase-treated mice (Fig. 4B).

For thioredoxin, IgE-dependent basophil degranulation was induced with all three concentrations, significantly increased only in 1 µg-sensitised group in comparison to controls. Similarly, immunisation with 1 µg and 5 µg arginine kinase induced significantly high IgE-depended basophil degranulation compared to controls (Fig. 4C).

### Cytokine production in sensitised mice

**Figure 5:**
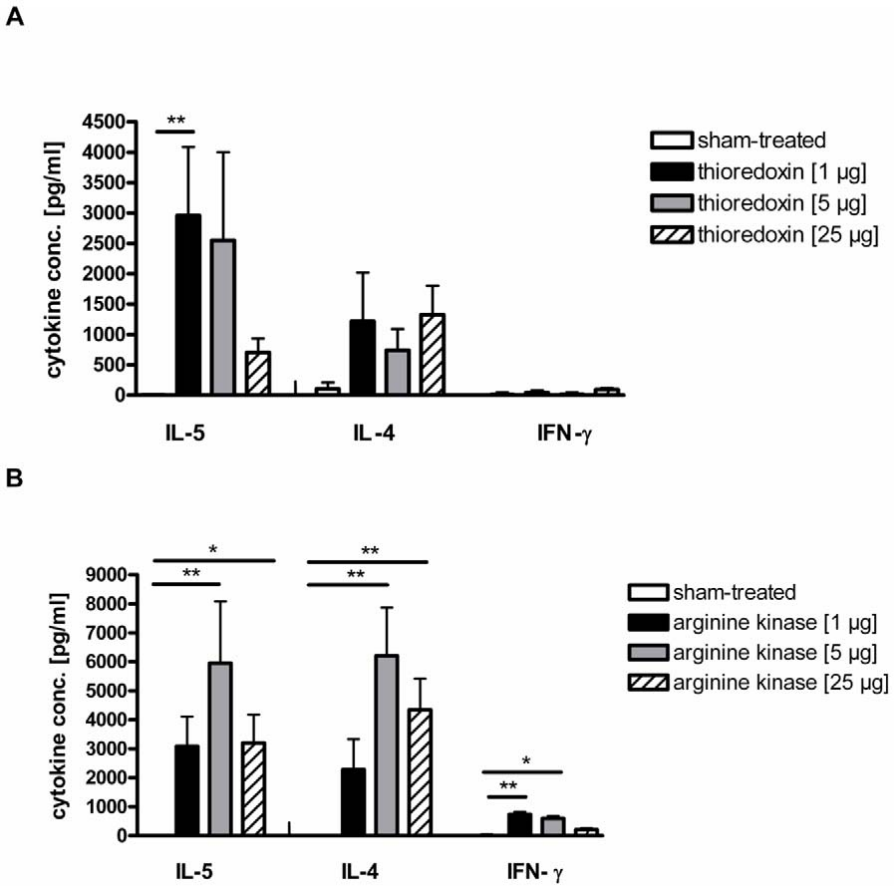
Cellular immune responses. IL-5, IL-4 and IFN-γ levels in supernatants of restimulated spleen cell cultures of mice, treated with 1 µg (black bars), 5 µg (grey bars) or 25 µg (striped bars) of (A), thioredoxin or (B), arginine kinase, or sham-treated (white bars). Groups were compared to sham-treated controls. *p<0.05, **p<0.01.

Both arginine kinase and thioredoxin induced strong cellular responses with high IL-5 and IL-4 levels in restimulated spleen cell cultures, while IFN-γ was induced only by arginine kinase. Th2 cytokine production in splenocytes of thioredoxin-treated mice showed significantly induced IL-5 levels in 1 µg-sensitised groups, compared to sham-treated mice. Thioredoxin-specific IL-4 was increased in all treated groups, in comparison to controls. Thioredoxin did not induce IFN-γ (Fig. 5A). Arginine kinase showed similar high IL-5 levels for all three sensitisation concentrations like thioredoxin. In particular, application of 5 µg arginine kinase showed the highest IL-5 and IL-4 production. Sensitisation with 5 µg or 25 µg arginine kinase induced significantly increased IL-4 levels compared to sham-treated group. Moreover, a low production of IFN-γ was measured in arginine kinase-treated mice, which reached significant IFN-γ levels in 1 µg and 5 µg treated mice compared to controls (Fig. 5B).

### Proliferative response of stimulated lymphocytes

Thioredoxin restimulated splenocytes showed an increased proliferation response (SI: 4) for all 3 treatment groups. Immunisation with 25 µg thioredoxin provoked a significantly induced proliferation compared to sham-treated mice.

**Figure 6:**
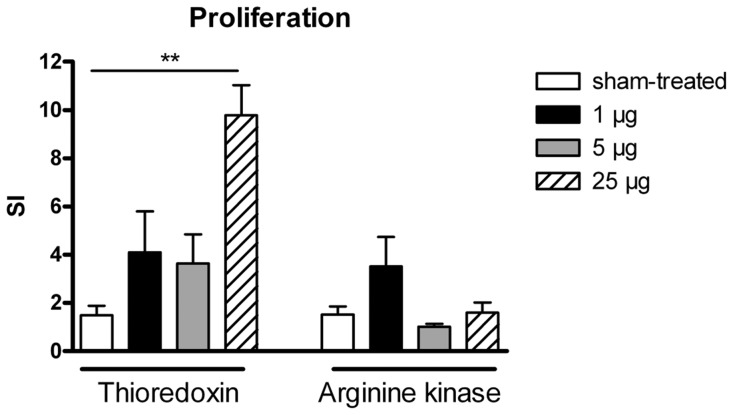
In vitro splenocyte stimulation. Stimulation index [SI] of thioredoxin or arginine kinase restimulated spleen cell cultures of mice, treated with 1 µg (black bars), 5 µg (grey bars) or 25 µg (striped bars) antigen, compared to sham-treated controls (white bars). **p<0.01.

Arginine kinase-sensitised mice showed cellular responses only in the group with 1 µg treatment concentration, not with 5 µg or 25 µg, in comparison to controls (Fig. 6).

## Discussion

The IgE immunoblot experiments probing indoor allergic patients' sera with extracts from adult moths confirmed previous results on larval extracts [12] and showed IgE positivity in 48% of the sera of indoor allergic patients, therefore moths have to be considered a relevant source of allergens.

The complex and only partially resolved immunoblot band patterns show that there are numerous moth IgE antigens. Although many patients without mite allergy have IgE against moth components, the *in vitro* reactivity is weaker than in mite positive patients. As some of these individuals may bind to cross-reactive carbohydrate determinants (CCDs) in the moth extracts, we do not know at this time how much mite-independent moth allergy could exist. Certainly, the allergens cross-reactive between moth and mite may contribute to this enhanced IgE reactivity. An additional phenomenon is that the mite and animal dander allergic patients together with the seafood allergic patients show the highest levels of IgE binding to moths. It appears as if these polysensitised patients show a stronger atopic predisposition and higher IgE levels. So far we do not know what is the contribution of moth allergens in the sensitisation of these patients.

IgE screening of a *P. interpunctella* cDNA library produced a number of immunopositive clones mostly encoding thioredoxin. Testing the moth positive sera against recombinant thioredoxin gave a positive reaction in 5% of all examined allergic patients, 8% of moth positive patients, whereas recombinant Plo i 1 tested positive in 14% of all sera, 25% of moth positive sera. The data show that thioredoxin (now named Plo i 2) is a minor allergen, however, the majority of those with positive reactivity showed a high level of thioredoxin-specific IgE. The prevalence of thioredoxin reactivity was highest in the group of indoor allergic patients reacting with mites and animal dander.

Recombinant thioredoxin like arginine kinase induced a Th2-biased immune response with significantly enhanced IgE and IgG1 antibody levels at the humoral site as well as a marked upregulation of IL-5 and IL-4 cytokines on the cellular site. Moreover, significantly enhanced IgE-mediated basophil degranulation with even 1 µg allergen confirmed the clinical relevant Th2-biased immune response of thioredoxin as well.

Thioredoxin is a truly ubiquitous protein with pleiotropic activities found both in eukaryotes and prokaryotes. It had been identified as an allergen in wheat and maize [19], in fungi [20], [21], and in this study for the first time in a representative from the animal kingdom strengthening its denomination as a member of a novel pan-allergen family [20] like the profilins [28].

Finally, we have noted that under certain conditions thioredoxins may also diminish the allergic response [29]. Treatment of commercial wheat allergen extract with reduced wheat thioredoxin, thioredoxin reductase, and NADPH reduced its allergenic potency, measured in a dog skin test model [30]. Similarly, thioredoxin reduced the allergenicity of β-lactoglobulin, the major bovine milk allergen [31]. In comparison to wild-type mice, transgenic mice overexpressing human thioredoxin showed a diminished mast cell histamine release in response to 2,4-dinitrophenylated bovine serum albumin [32]. Apoptosis can be induced in human bronchial epithelial cells by house dust mite-stimulated eosinophils. This effect is weaker when the epithelial cells overexpress human thioredoxin [33]. Further studies will have to show whether moth thioredoxin might also have any moderating activity on allergic responses similar to those reported for wheat and human thioredoxins.
